# Supplementary material for: Effect of internet-delivered cognitive behavioral therapy on insomnia in convalescent patients with COVID-19: Protocol for a systematic review and meta-analysis
Source: PLoS One. 2022 Jun 14;17(6):e0269882. doi: 10.1371/journal.pone.0269882 (PMC9197064; doi:10.1371/journal.pone.0269882)
Supplement: S1 Fig — (DOCX) [file pone.0269882.s002.docx]

## Screening

## Included

## Eligibility

Records identified through database searching
(n = )

## Identification

Additional records identified through other sources
(n = )

Total records identified
(n = )

Records screened by titles and abstracts

(n = )

Records excluded
(n = )

Full-text articles assessed for eligibility
(n = )

Full-text articles excluded, with reasons
(n = )

Conference abstract

(n = )

Inappropriate outcomes (n = )

Incomplete or no data

(n = )

Non-randomized controlled trial (n = )

Studies included in quantitative synthesis (meta-analysis)
(n = )

Duplicate records removed
(n = )

**Figure 1.** **Flow diagram of study selection process.**
