# Supplementary material for: Effect of internet-delivered cognitive behavioral therapy on insomnia in convalescent patients with COVID-19: Protocol for a systematic review and meta-analysis
Source: PLoS One. 2022 Jun 14;17(6):e0269882. doi: 10.1371/journal.pone.0269882 (PMC9197064; doi:10.1371/journal.pone.0269882)
Supplement: Attachment — Submitted filename: Response to Reviewers.docx [file pone.0269882.s003.docx]

Dear Editor,

We would like to resubmit the revised manuscript entitled “Effect of Internet-delivered cognitive behavioral therapy on insomnia in convalescent patients with COVID-19: protocol for a systematic review and meta-analysis (PONE-D-21-40745)” for consideration by PLOS ONE. We would like to thank the reviewers for thoroughly reviewing our manuscript and making many thoughtful comments. We were very pleased to see that reviewers recognized the novelty and potential significance of our work. We have added significant new contents, described in detail below, and revised the manuscript to address reviewers’ comments. In the Revised Manuscript with Track Changes, the contents we added are marked in green and the contents we deleted are marked in red. Here are our point-by-point responses:

Yan Chen

Institute of Chronic Disease Risks Assessment, School of nursing and health,

Henan University

Kaifeng, Henan, China

E-mail address: chenyan8251@126.com

March 23, 2022

**Journal Requirements:**

1. When submitting your revision, we need you to address these additional requirements.

Please ensure that your manuscript meets PLOS ONE's style requirements, including those for file naming. The PLOS ONE style templates can be found at https://journals.plos.org/plosone/s/file?id=wjVg/PLOSOne_formatting_sample_main_body.pdf and <https://journals.plos.org/plosone/s/file?id=ba62/PLOSOne_formatting_sample_title_authors_affiliations.pdf>

Answer:

We have revised our manuscript to meet meets PLOS ONE's style requirements.

2. In your Data Availability statement, you have not specified where the minimal data set underlying the results described in your manuscript can be found. PLOS defines a study's minimal data set as the underlying data used to reach the conclusions drawn in the manuscript and any additional data required to replicate the reported study findings in their entirety. All PLOS journals require that the minimal data set be made fully available. For more information about our data policy, please see http://journals.plos.org/plosone/s/data-availability.

Upon re-submitting your revised manuscript, please upload your study’s minimal underlying data set as either Supporting Information files or to a stable, public repository and include the relevant URLs, DOIs, or accession numbers within your revised cover letter. For a list of acceptable repositories, please see http://journals.plos.org/plosone/s/data-availability#loc-recommended-repositories. Any potentially identifying patient information must be fully anonymized.

Important: If there are ethical or legal restrictions to sharing your data publicly, please explain these restrictions in detail. Please see our guidelines for more information on what we consider unacceptable restrictions to publicly sharing data: http://journals.plos.org/plosone/s/data-availability#loc-unacceptable-data-access-restrictions. Note that it is not acceptable for the authors to be the sole named individuals responsible for ensuring data access.

We will update your Data Availability statement to reflect the information you provide in your cover letter.

Answer:

All relevant data from this study will be made available upon study completion.

3. Your ethics statement should only appear in the Methods section of your manuscript. If your ethics statement is written in any section besides the Methods, please delete it from any other section.

Answer:

Thank you for your comments. We have moved the ethics statement to the Methods section of the manuscript as required. Please review it again.

4. We note that this manuscript is a systematic review or meta-analysis; our author guidelines therefore require that you use PRISMA guidance to help improve reporting quality of this type of study. Please upload copies of the completed PRISMA checklist as Supporting Information with a file name “PRISMA checklist”.

Answer:

We have used PRISMA guidelines to help improve the reporting quality of this study. At the same time, we have uploaded a copy of the completed PRISMA checklist as supporting information.

5. Please review your reference list to ensure that it is complete and correct. If you have cited papers that have been retracted, please include the rationale for doing so in the manuscript text, or remove these references and replace them with relevant current references. Any changes to the reference list should be mentioned in the rebuttal letter that accompanies your revised manuscript. If you need to cite a retracted article, indicate the article’s retracted status in the References list and also include a citation and full reference for the retraction notice.

Answer:

We have carefully examined the reference list to ensure its completeness and correctness.

Comments to the Author

Number 1:

1. Does the manuscript provide a valid rationale for the proposed study, with clearly identified and justified research questions?

The research question outlined is expected to address a valid academic problem or topic and contribute to the base of knowledge in the field.

Reviewer #1: Partly

Answer:

Thank you for your comments.

Number 2:

2. Is the protocol technically sound and planned in a manner that will lead to a meaningful outcome and allow testing the stated hypotheses?

The manuscript should describe the methods in sufficient detail to prevent undisclosed flexibility in the experimental procedure or analysis pipeline, including sufficient outcome-neutral conditions (e.g. necessary controls, absence of floor or ceiling effects) to test the proposed hypotheses and a statistical power analysis where applicable. As there may be aspects of the methodology and analysis which can only be refined once the work is undertaken, authors should outline potential assumptions and explicitly describe what aspects of the proposed analyses, if any, are exploratory.

Reviewer #1: Yes

Answer:

Thank you for your comments.

Number 3:

3. Is the methodology feasible and described in sufficient detail to allow the work to be replicable?

Descriptions of methods and materials in the protocol should be reported in sufficient detail for another researcher to reproduce all experiments and analyses. The protocol should describe the appropriate controls, sample size calculations, and replication needed to ensure that the data are robust and reproducible.

Reviewer #1: Yes

Answer:

Thank you for your comments.

Number 4:

4. Have the authors described where all data underlying the findings will be made available when the study is complete?

The PLOS Data policy requires authors to make all data underlying the findings described in their manuscript fully available without restriction, with rare exception, at the time of publication. The data should be provided as part of the manuscript or its supporting information, or deposited to a public repository. For example, in addition to summary statistics, the data points behind means, medians and variance measures should be available. If there are restrictions on publicly sharing data—e.g. participant privacy or use of data from a third party—those must be specified.

Reviewer #1: Yes

Answer:

Thank you for your comments.

Number 5:

5. Is the manuscript presented in an intelligible fashion and written in standard English?

PLOS ONE does not copyedit accepted manuscripts, so the language in submitted articles must be clear, correct, and unambiguous. Any typographical or grammatical errors should be corrected at revision, so please note any specific errors here.

Reviewer #1: No

Answer: Thank you very much for your valuable comments. We have carefully polished the language of the manuscript to make it readable and standard.

Number 6:

6. Review Comments to the Author

Please use the space provided to explain your answers to the questions above and, if applicable, provide comments about issues authors must address before this protocol can be accepted for publication. You may also include additional comments for the author, including concerns about research or publication ethics.

You may also provide optional suggestions and comments to authors that they might find helpful in planning their study.

(Please upload your review as an attachment if it exceeds 20,000 characters)

Answer:

Thank you.

Number 7:

Reviewer #1: The article by Chen et al. presented the study design of a metanalysis and systematic research to evaluate the effects of internet-delivered cognitive behavioral therapy on insomnia in COVID-19 patients in convalescence. Methods are sounds, however there are some minor concerns:

In the abstract section:

1) “In addition. I2 and Cochrane will be used for heterogeneity assessment.” I don’ understand the full stop just after “In addition”. AMEND

Answer:

Thank you very much for your careful review. We have corrected the writing errors caused by our carelessness.

In the introduction section:

2) “and despair [9, 10], Even” comma or full stop? AMEND.

Answer:

We have changed "comma" to "full stop" here.

3) “Insomnia is defined as the subjective perception of difficulties in falling asleep, maintaining asleep, and early morning awakening at least three times per week for at least 3 months^[13]^.” Imsonnia not is a subjective perception and the article that is cited does not define it as a subjective perception. Please correct.

Answer:

Thank you for your valuable comments. We have carefully checked the quoted articles and made the following changes to the definition of insomnia:

Insomnia is defined as difficulties in falling asleep, maintaining asleep, or waking up too early at least three times per week for at least 3 months [13] and is one of the most common problems experienced by convalescent patients with COVID-19 [14].

4) “Till now, Previous studie” comma or full stop? AMEND.

Answer:

Thank you for your careful review. It is correct to use comma here, and we have changed the description of this sentence. Please review it again.

5) “But a recent study found that Internet cognitive”. Use “However” instead of “But”.

Answer:

Thank you for your suggestion. We have replaced "But" with "However".

6) There are many examples of internet-delivered therapy for neurological diseases and a recent review of the literature thoroughly presented them. I suggest to briefly discuss it in the introduction section (PMID: 34018047).

Answer:

Thank you for your constructive suggestions. We refer to a recent review and add the following contents to make the manuscript more comprehensive:

As a kind of digital therapy, Internet-delivered cognitive behavioral therapy provides patients with evidence-based treatment interventions, which are driven by software to prevent, manage, or treat medical disorders or diseases [31]. It can be used independently or in concert with medications, devices, or other therapies [31]. Internet-delivered cognitive behavioral therapy, as a promising method to solve psychotherapy obstacles [32], can overcome several limits related to traditional clinical practice [33].

The article could improve with a revision by an english native speaker.

Answer:

We have carefully polished the language of the manuscript to make it more professional and more suitable for publication.

7. PLOS authors have the option to publish the peer review history of their article (what does this mean?). If published, this will include your full peer review and any attached files.

If you choose “no”, your identity will remain anonymous but your review may still be made public.

Do you want your identity to be public for this peer review? For information about this choice, including consent withdrawal, please see our Privacy Policy.

Reviewer #1: No

Thank you for your consideration of our manuscript.

Yours sincerely,

Yan Chen
