# Supplementary material for: Effect of internet-delivered cognitive behavioral therapy on insomnia in convalescent patients with COVID-19: Protocol for a systematic review and meta-analysis
Source: PLoS One. 2022 Jun 14;17(6):e0269882. doi: 10.1371/journal.pone.0269882 (PMC9197064; doi:10.1371/journal.pone.0269882)
Supplement: Attachment — Submitted filename: Response to Reviewers.docx [file pone.0269882.s004.docx]

Dear Editor,

We would like to resubmit the revised manuscript entitled “Effect of Internet-delivered cognitive behavioral therapy on insomnia in convalescent patients with COVID-19: protocol for a systematic review and meta-analysis (PONE-D-21-40745R1)” for consideration by PLOS ONE. We would like to thank the reviewers for thoroughly reviewing our manuscript and making many thoughtful comments. We were very pleased to see that reviewers recognized the novelty and potential significance of our work. We have added significant new contents, described in detail below, and revised the manuscript to address reviewers’ comments. In the Revised Manuscript with Track Changes, the contents we added are marked in green and the contents we deleted are marked in red. Here are our point-by-point responses:

Yan Chen

Institute of Chronic Disease Risks Assessment, School of nursing and health,

Henan University

Kaifeng, Henan, China

E-mail address: chenyan8251@126.com

April 6, 2022

**Journal Requirements:**

(1) The date of the search run was the 30th of July in the manuscript, while the PROPSERO entry was the 11th of December. Please, can you clarify when the search was run?

Answer: Thank you very much for your careful review. The search run in the registration protocol is the 30th of July. However, given the prevalence of COVID-19 epidemic, we consider that there may be more published articles that meet the criteria of the study. To expand the search scope, the search run in the manuscript is the 11th of December. Combined with the your comments, we have changed the retrieval time to the 30th of July in the manuscript to make it consistent with the research protocol.

(2) Please, in the Methods section, can you clarify whether the data have already been collected and analyzed?

Answer: Thank you for your valuable comments. Till now, the data have already been collected and analyzed. We have clarified it in the Methods section.

(3) Ideally, Study Protocols should be written in the present tense. We noted that the tense used in the manuscript is the past tense. Could you please correct accordingly?

Answer: Thank you very much for your valuable comments. We have carefully modified the tense used in the manuscript.

(4) We noted significant overlap between your work and the article titled ". Efficacy and safety evaluation of bright light therapy in patients with post-stroke insomnia: a protocol of systematic review and meta-analysis" (Lei H et al. Medicine. 2021;100:50(e27937)). Please, can you comment on this?

Answer: Thanks for your careful review. As you pointed out, the study entitled "Efficacy and safety evaluation of bright light therapy in patients with post-stroke insomnia: a protocol of systematic review and meta-analysis (PMID: 34918641) has some similarities in the selection of outcome indicators with our study. However, after careful reading, we find great differences in the design of the two studies. Firstly, the research objects are different. The research objects of our study are COVID-19 patients in rehabilitation, while the above study (PMID:34918641) focuses on the insomnia population with stroke. Although the two studies all use insomnia as an outcome indicator, insomnia in stroke patients may be caused by nervous system damage, and our study is aimed at insomnia in COVID-19 rehabilitation patients. Secondly, the intervention methods are different. Our study uses Internet-delivered cognitive behavioral therapy. Cognitive behavioral therapy, as an evidence-based option, has been found to effectively improve sleep outcomes [1, 2] and its therapeutic effect is equivalent to or better than that of drug therapy [3]. However, the above study (PMID:34918641) selects bright light therapy (Typically, patients are instructed to be exposed to bright light at a constant distance every day[4]).

**References**

[1] Trauer JM, Qian MY, Doyle JS, Rajaratnam SM, Cunnington D. Cognitive Behavioral Therapy for Chronic Insomnia: A Systematic Review and Meta-analysis. Ann Intern Med. 2015 4; 163(3):191-204. https://doi.org/10.7326/M14-2841 PMID: 26054060

[2] Irwin MR, Cole JC, Nicassio PM. Comparative meta-analysis of behavioral interventions for insomnia and their efficacy in middle-aged adults and in older adults 55+ years of age. Health Psychol. 2006; 25(1):3-14. https://doi.org/10.1037/0278-6133.25.1.3 PMID: 16448292

[3] Smith MT, Perlis ML, Park A, Smith MS, Pennington J, Giles DE, Buysse DJ. Comparative meta-analysis of pharmacotherapy and behavior therapy for persistent insomnia. Am J Psychiatry. 2002; 159(1):5-11. https://doi.org/10.1176/appi.ajp.159.1.5 PMID: 11772681

[4] van Maanen A, Meijer AM, van der Heijden KB, Oort FJ. The effects of light therapy on sleep problems: A systematic review and meta-analysis. Sleep Med Rev. 2016; 29:52-62. http://doi.org/ 10.1016/j.smrv. PMID: 26606319
