# Supplementary material for: Effect of internet-delivered cognitive behavioral therapy on insomnia in convalescent patients with COVID-19: Protocol for a systematic review and meta-analysis
Source: PLoS One. 2022 Jun 14;17(6):e0269882. doi: 10.1371/journal.pone.0269882 (PMC9197064; doi:10.1371/journal.pone.0269882)
Supplement: Attachment — Submitted filename: Response to Reviewers.docx [file pone.0269882.s005.docx]

Dear Editor,

We would like to resubmit the revised manuscript entitled “Effect of Internet-delivered cognitive behavioral therapy on insomnia in convalescent patients with COVID-19: protocol for a systematic review and meta-analysis (PONE-D-21-40745R1)” for consideration by PLOS ONE. We would like to thank the reviewers for thoroughly reviewing our manuscript and making many thoughtful comments. We were very pleased to see that reviewers recognized the novelty and potential significance of our work. We have added significant new contents, described in detail below, and revised the manuscript to address reviewers’ comments. In the Revised Manuscript with Track Changes, the contents we added are marked in green and the contents we deleted are marked in red. Here are our point-by-point responses:

Yan Chen

Institute of Chronic Disease Risks Assessment, School of nursing and health,

Henan University

Kaifeng, Henan, China

E-mail address: chenyan8251@126.com

April 17, 2022

**Journal Requirements:**

1) we noted that the data analysis for this study in now completed, as you reported in the Methods section. Study Protocol are articles type for which data collection and analysis cannot be completed at the moment of submission. Please, remove the sentence from the Methods.

Answer: Thank you for your valuable comments. We have deleted the sentence "Till now, the data have already been collected and analyzed." and further clarified it in the Methods.

2) Please, edit your manuscript with the present or future tense in order to make it conform to the Study Protocol criteria. Study protocol present the proposal of a study, and the manuscript should read as something you are proposing to do.

Answer: Thank you very much for your valuable comments. We have carefully modified the tense used in the manuscript.

3) The search date between the manuscript and the PROSPERO entry are still inconsistent. As you extended the search strategy to the 11th of December, please report this into the main text of the manuscript.

Answer: Thank you very much for your careful review. The search run in the registration protocol is the 30th of July. However, given the prevalence of COVID-19 epidemic, we consider that there may be more published articles that meet the criteria of the study. Hence, the search run in the manuscript is the 11th of December so as to expand the search scope. In consideration of your comments, we have clarified it in the main text of the manuscript.

4) We appreciate your response on the differences between your study and Lei H et al. Medicine. 2021;100:50(e27937). However, we feel you did not provide sufficient explanation on why the search strategies are similar. Please, clarify this point."

Answer: Thanks for your careful review. As you pointed out, the study entitled "Efficacy and safety evaluation of bright light therapy in patients with post-stroke insomnia: a protocol of systematic review and meta-analysis (PMID: 34918641) has some similarities with our study in search strategies.

After careful review, we found that the similarity is mainly due to two reasons. Firstly, this study (PMID: 34918641) is different from our study in the research object, but it is the same in the analysis of the outcome index, namely insomnia. Secondly, both the study (PMID:34918641) and our study use subject words [Sleep Initiation and Maintenance Disorders[Mesh]] combined with entry terms [(Disorders of Initiating and Maintaining Sleep) OR (Disorders of Initiating and Maintaining Sleep) OR (Early Awakening) OR (Awakening, Early) OR (Nonorganic Insomnia) OR (Insomnia, Nonorganic) OR (Primary Insomnia) OR (Insomnia, Primary) OR (Transient Insomnia) OR(Insomnia, Transient) OR (Rebound Insomnia) OR (Insomnia, Rebound) OR (Secondary Insomnia) OR (Insomnia, Secondary) OR (Sleep Initiation Dysfunction) OR (Dysfunction, Sleep Initiation) OR (Dysfunctions, Sleep Initiation) OR (Sleep Initiation Dysfunctions) OR (Sleeplessness) OR (Insomnia Disorder) OR (Insomnia Disorders) OR (Insomnia) OR (Insomnias) OR (Chronic Insomnia) OR (Insomnia, Chronic) OR (Psychophysiological Insomnia) OR (Insomnia, Psychophysiological)] to retrieve the PubMed. This scientific approach can help to obtain more comprehensive search results. Therefore, due to the same outcome index and search method, the two studies are similar in search strategies.

Thank you for your consideration of our manuscript.

Yours sincerely,

Yan Chen
